# Some Points in Diphtheria

**Published:** 1881-04

**Authors:** C. J. Lewis


### Article II.

Some Points in Diphtheria. By C. J. Lewis, m. d. Read before the West Chicago Medical Society, February 14th, 1881.

*Mr. President, Ladies and Gentlemen:*—That inexorable governess, Fashion, rules no less in medicine than in the æsthetic arts, in dress or in religion. She has formulated dogma for the votaries of Hygeia to amplify by their life work, with a special request to uplift their "Brazen Serpent" with a zeal and energy second to the devotees of no creed, of no religion, nor of any philosophic bias. Thus we have had a school of humoralists, of solidists, and lately, of vitalists.

For the last twenty to twenty-five years, the creed for diphtheria has been its bacterial origin. This dogma has either been more enthusiastically indorsed or passively acquiesced in by the profession, than any other creed that has ever been formulated in medicine. Upon this substructure, composed of spores moving hither and thither with remarkable alertness logically, is constructed the doctrine of contagion and sepsis. If the basic substance of this substructure—pseudo-membrane—is inadequate to build it up *from within*, and there should arise good reasons for believing that it is largely formed by accretion, as it seems the experiments of Drs. H. C. Wood and Henry F. Formad indicate, then the theory of contagion and sepsis falls prostrate at our feet.

Jacobi says of the poison of diphtheria, "that it behaves in the same manner as the acarus scabiei: that this poison is certainly transmitted by spoons, glasses, handkerchiefs and towels used by the patients. * * Moreover, the nature of the affection is still little understood, and the question of a local or a general beginning of many individual cases of the disease still remains a mooted point." Whilst Jacobi is somewhat skeptical as to the germ origin, he, in the above quotations, expresses a belief that the disturbance set up in the human organism by diphtheria is the direct result of an entity—a poison that can be seen, handled, and, as many can testify concerning the acarus, be felt. According to this theory, this mythical something, which is so restless, so turbulent, and, withal, so fleet of wing, after multiplying a million fold diffuses itself in or becomes intermixed with the atmosphere, and from thence is lodged upon clothing, sores, or furniture in the adjacent areas of its first attack, as well as to pierce the mucous membrane of the mouth and fauces as the bacterate air is respired, and soon becomes commingled with the blood—its natural abode—where it generates other untold millions, resulting in the so-called blood-poisoning.

In this manner the poison is absorbed into and contaminates the system from the lungs, a sore on the skin, or, preferably, the mucous membrane of the mouth and fauces. But what is singular of this *poison*, this *entity*, this *thing of dread*, is, to quote Jacobi again—"that the diphtheritic membranes which are swallowed are rendered innocuous by the 'gastric fluids.'"

This intelligence and wisdom of the "gastric fluids," to say the least, is remarkable in that it "renders innocuous" the poison of diphtheria, having its nidus in the membrane which the patient may swallow—while such a guardian angel stands by, as a pastime; when the same "fluid" fails to "render innocuous" strychnia, arsenic, veratrum, or any of the other known poisons. I am at a loss to know how this author can see the diphtheritic process enter the blood by the lungs, through a sore, or an abrasion, and utterly fail to observe its entrance into the vascular system by the open mouth of the absorbents in the primæ viæ. Relying upon my own observations and the experiments of recent observers, I am of opinion that diphtheria is not produced by germs, nor do I believe it to be contagious.

From what has now been said, I am prepared to turn the powerful enginery constructed by Jacobi for the express purpose of demolishing the arguments of the bacterial philosophers, against the framers, upholders and promoters of the contagion theory, namely: "The constant repetition of the same statements does not render them any the sounder." * * * "For it is certainly very apparent, here, too, that the nature of the diphtheritic poison has been postulated in the interest of the contagion theory."

To me, diphtheria is a pyrexia,—a disease having a special predilection for evolving its inflammatory products in, or upon, the mucous membrane of all of the upper portions of the respiratory tract. This pyrexia or inflammatory fever is, in my judgment, closely allied to rheumatism ; but having more of adynamia than rheumatism, and, when epidemic, it followed periods when rheumatism had prevailed. Mackenzie says that "The contagion lives under ordinary atmospheric conditions, but it is probable that dampness favors its development." Damp weather is likewise thought to favor the development of rheumatism. Then, again, pain is a prominent symptom in diphtheria. The pain caused in the fauces by attempts to swallow liquids is out of all proportion to the apparent local lesion. About four-fifths of my cases had as a first symptom, *soreness and stiffness of the muscles of the neck.* Mackenzie says there is at the outset stiffness of the neck ; Dr. A. B. Woodward, of Pennsylvania, says that "almost every case has been taken with a stiff neck ;" and a Dr. Hitchman, of New York, mentions stiffness of the neck as a symptom. These three are all whom I have been able to find, who give soreness and stiffness of the muscles of the neck as a symptom in diphtheria.

But the action of the diptheritic process upon the heart and blood vessels is almost a perfect prototype of the rheumatic process. In grave cases, the heart beats in diphtheria very fast at first, then slowing and intermitting, towards the close in fatal cases, it beats very feeble and fast. This is very like the action of the heart in rheumatism or aconite poisoning. Mackenzie says that the pulse is very weak and compressible, and often either exceptionally rapid or exceptionally slow, while the first sound of the heart is muffled and devoid of tone." But Jacobi has so aptly described the behavior of the heart under the diphtheritic poison, which is such a direct parallelism to rheumatism, that I beg your indulgence in quoting the paragraph, namely : "The result, of course is considerable weakness of its muscular tissue, evidenced by the formation of local (Beverly Johnson) thrombi ; general sluggishness of the circulation, dyspnœa, muffled heart sounds, a cool and pale skin, and sudden death, preceded by a very feeble and frequent, sometimes, however, by a very slow pulse. Aside from this, there are actual cases of endocarditis during the course of diphtheria, or convalescence therefrom. It affects especially the valves, and among them particularly the mitral. It is characterized by high fever, precordial pain, attacks of syncope, and a systolic murmur." So we see that both of the above cliniciens undeniably hold that the heart failure in diphtheria is primarily due to septic forces. It seems to me, however, that if we would be in close accord with the clinical study of diphtheria, that this adynamic state has a more rational explanation in the rheumatoid condition of the heart, the anæsthesia of the vaso-motor nerves, and the consequent hyperæmia, especially of the uriniferous tubules of the kidneys, than in a specific sepsis or blood-poisoning. For it can but be hurtful to the granular spot, or morphological unit, of each and every cell composing our bodies to have blood offered to it for nutrition, which is heavily laden with detritus, resulting from the vastly diminished exhalation, or lessened functional activity of the hyperæmic state of the kidneys, skin, and lungs.

There is local hyperæmia in the upper respiratory tract, evidenced by the deep livid hue of the mucous membrane, which, on account of the anatomical relation of the blood vessels, is made very apparent. Through the inflammatory process the dilatation of the walls of these vessels occurs on the side where there is the least resistance allowing in this manner a greater freedom for exosmosis towards the mucous membrane of the inflammatory *precincts*, namely, the *diphtheritic membrane.* The redness of the fauces is soon superseded with lividity, at which time the accompanying symptoms make it possible to diagnosticate diphtheria. This *livid color* of the faucial mucous membrane, according to my observation, is characteristic of diphtheria before the appearance of the membrane. Dr. Goss, of Tanner fame, in describing diphtheria, says, " The fauces look inflamed, they are of a dark mahogany color." And a Dr. E. G. Jones, in a journal, mentions lividity of the mucous membrane of the fauces, tonsils, and pharynx as a symptom. Jacobi says there is hyperæmia.

### POINTS.

The basic substance of the diphtheritic membrane is eliminated from the blood, if diphtheria is produced by sewer gas or decomposing organic matter. But there seems to be good reason for believing that the pseudo-membrane is largely formed by accretion, and wholly so, so far as it is composed of germs. Then a failure to prove that diphtheria springs *de novo* from bacteriæ, renders the theory of contagion and sepsis untenable.

Diphtheria is a pyrexia. It is a rheumatoid adynamia, with anæsthesia of the vaso-motor nerves, and consequent hyperæmia of the uriniferous tubules and capillaries, followed by a diminished exhalation, or functional activity of the kidneys, skin and lungs. The great emunctories thus lagging in their scavenger work, there comes coincidently over the person a malaise.

Lividity of the mucous membrane of the fauces, tonsils, and pharynx, with soreness, swelling and stiffness of the muscles of the neck, are marked symptoms in diphtheria. The diphtheritic membrane, being an exudate to the surface by the outward free exosmosis, becomes very soon a nidus for bacteriæ.

I will here describe two cases; one, a so-called blood-poisoning, the other, a case of the sthenic type.

At the bedside of a well-nourished boy, aged about 2½ years, in mid winter, I find him lying on his back, with intense pallor of the face and lips; large drops of cold sweat on the forehead; the hands and surface of the body yielding a cold and clammy feel; pulse 180 per minute; exceedingly feeble; swelling of the glands of the neck, with soreness and stiffness of the lateral muscles of the neck; tumescence and lividity of the mucous membrane of the pharynx, tonsils and fauces, and soreness of throat. This patient did not develop a membrane in this illness. The pupils were widely dilated, and he was profoundly indifferent to all that was going on around him, excepting that he complained of pain when efforts were made to move his head. This little patient had come from comparative good health to this condition in about twenty hours after having been unduly exposed to cold.

The sthenic case—an adult, was delivering coal from the cars on a cart to families, a November day, when the weather was, according to the paragraphers—a blizzard. While loading he sweated, and cooled when riding. He complained nearly all day of having chilly sensations, followed by flushes of heat, and as the day wore on, his neck stiffened and his throat became sore. At night his throat was parched, was thirsty, and restless from "fever." The following day finds him with all the symptoms intensified. When I arrived at 5 P. M., his skin was dry, pulse 120 and hard, face flushed, neck stiff, with but little swelling of the glands of the neck. The tonsils and back of the pharynx were covered with pseudo-membrane, and the adjacent mucous membrane was of a livid color. The tongue was heavily coated with fur. He complained of frequent micturition.

*Treatment.*—The arm of the physician is not much better than though it were palsied, if he is unable to bear in his open hand the needed remedies fresh from his armamentarium to either dispel from, or to neutralize in his patient the morbific forces that oppress him. After settling in his mind what these forces probably are, he systematizes the indications. The administration of remedial agents without well ordered plans would be tentative—empirical.

The first indications in diphtheria are to lessen the inflammatory engorgement of the mucous membrane in the upper respiratory tract and kidneys; and, second, to restore the functional activity of the skin and kidneys.

A very good plan to meet the first indications, and in my hands a very successful one, is to lessen the irritability with opium, to arrest the tendency to dilatation of the capillaries with small doses of belladonna and aconite, and to modify the epithelium with small doses of potassic chlorate. These drugs may be combined for a child aged 2 to 3 years, as follows:

℞. Tr. Aconit. rad.......................... gtt. x.

Tr. Belladonn............................ gtt. xx.

Tr. Opii deod............................ gtt. xxx.

Potass. chlorat.......................... ʒi.

Aq. dist. ..................... ad ... ℥iv

M. S. Give a teaspoonful every hour or two.

From 10 to 15 doses of this prescription would be given in 24 hours.

In addition to the above I usually give something like the following:

℞. Sod. salicylat.............................. ʒi.

Spt. nit. dulc................................ ℥ij.

Syr. tolu...................................... ʒxiv.

M. S. Give a teaspoonful every two hours.

Locally on the swelled glands of the neck I apply Ol. camph. U. S. P.

Jacobi, on page 197, relates having treated what he called "diphtheritic fever" successfully with salicylic acid and bicarbonate of sodium. This points very strongly to a rheumatoid state.

I treat the sthenic and asthenic forms of diphtheria alike; excepting that I maintain an increased opium influence in the asthenic variety for from twenty-four to thirty-six hours.

The improvement in the condition of the patient is noted by the slowing of the pulse; by the gentle and uniform moistening of the skin; a thinning, and the final removal of the exudate in from 30 to 50 hours; a gradual return of the arterial hue of mucous membrane of the fauces, tonsils and pharynx; relief from pain, diminished thirst and normal urine.

